# Treatment of patients with sepsis in a closed intensive care unit is associated with improved survival: a nationwide observational study in Japan

**DOI:** 10.1186/s40560-018-0322-8

**Published:** 2018-09-03

**Authors:** Takayuki Ogura, Yoshihiko Nakamura, Kunihiko Takahashi, Kazuki Nishida, Daisuke Kobashi, Shigeyuki Matsui

**Affiliations:** 1 Department of Emergency Medicine and Critical Care Medicine, Japan Red Cross Maebashi Hospital, Asahi-cho 3-21-36, Maebashi, Gunma 371-0014 Japan; 2 Department of Emergency and Critical Care Medicine, Faculty of Medicine, Fukuoka University, Fukuoka, Japan; 3 Department of Biostatistics, Nagoya University Graduate School of Medicine, Nagoya, Japan

**Keywords:** Intensive care unit, Sepsis, Intensivist, Mortality

## Abstract

**Background:**

The aim of this study is to investigate the association between treatment in a closed ICU and survival at discharge in patients with sepsis.

**Methods:**

This is a post hoc analysis utilizing data from the Japan Septic Disseminated Intravascular Coagulation study, including data from patients with sepsis from 2011 to 2013. Multiple logistic regression analysis was used to estimate the association between ICU policy and survival at discharge, and propensity score matching analysis was performed including the same covariates as a sensitivity analysis. Multiple linear regression analysis for the length of ICU stay in surviving patients was also performed with adjustments for the same covariates.

**Results:**

Two thousand four hundred ninety-five patients were analyzed. The median Acute Physiology and Chronic Health Evaluation (APACHE) II score was 22 [17–29], the median Sequential Organ Failure Assessment (SOFA) score was 9 [7–12], and the overall mortality was 33%. There were 979 patients treated in 17 open ICUs and 1516 patients in 18 closed ICUs. In comparison, the APACHE II score and SOFA scores were significantly higher in patients in closed ICUs (closed vs open = 23 [18–29] vs 21 [16–28]; *p* < .001, 9 [7–13] vs 9 [6–12]; *p* = 0.004). There was no difference in the unadjusted mortality (closed vs open; 33.1% vs 33.2%), but in multiple logistic regression analysis, treatment in a closed ICU is significantly associated with survival at discharge (odds ratio = 1.59, 95% CI [1.276–1.827], *p* = .001). The sensitivity analysis (702 pairs of the matching) showed a significantly higher survival rate in the closed ICU (71.8% vs 65.2%, *p* = 0.011). The length of ICU stay of patients in closed ICUs was significantly shorter (20% less).

**Conclusion:**

This Japanese nationwide analysis of patients with sepsis shows a significant association between treatment in a closed ICU and survival at discharge, and a 20% decrease in ICU stay.

**Electronic supplementary material:**

The online version of this article (10.1186/s40560-018-0322-8) contains supplementary material, which is available to authorized users.

## Background

Sepsis is life-threatening organ dysfunction caused by dysregulated host responses to infection, and septic shock is a subset of sepsis in which underlying circulatory and cellular/metabolic abnormalities are sufficiently profound to substantially increase mortality [1]. According to one study, there are more than 750,000 patients annually in the USA with sepsis [2] and the incidence is rising [3]. Septic shock remains lethal even with aggressive management, with a mortality of 20 to 30% [4].

Patients with sepsis are usually treated in the intensive care unit (ICU). Sepsis results from infection, and these patients often develop multiple organ-system failure. Aggressive management, including control of the infection source and support of failing organ-systems, is needed for optimal outcomes. In recent years, guidelines for the treatment of patients with sepsis have been published [5, 6], and intensivists play an important role in the care of these patients.

Intensivists improve patient outcome in the ICU [7], and the ICU organization model influences patient outcomes [8–10]. There are generally two staffing models for ICU treatment, including an “open organization model” and a “closed organization model.” In the open model, there is no intensivist consultation or elective intensivist consultation. Mandatory intensivist consultation is conducted in closed model ICUs [8], in which intensivist directs patient care [11] regardless of the time of day.

However, focusing on sepsis, to the best of our knowledge, the effect of the ICU organization model (open/closed) or directing of care by an intensivist on the mortality of patients is unknown and there are no studies to answer this important clinical question. We hypothesize that a closed ICU improves the outcome of patients with sepsis and septic shock, since a closed ICU is the highest-intensity physician staffing ICU model and intensivists direct care regardless of the time of day in the ICU. The aim of this study is to investigate the association between management in a closed ICU and survival at discharge of patients with sepsis. This is a nationwide study in Japan, including an analysis of clinical data regarding the severity of sepsis, pre-existing co-morbidities, the need for mechanical organ system support, and treatments given.

## Methods

### Patient selection

>This is a post hoc analysis utilizing the database from the Japan Septic Disseminated Intravascular Coagulation study (JSEPTIC DIC study) (University Hospital Medical Information Network Individual Case Data Repository, UMIN000012543, http://www.umin.ac.jp/icdr/index-j.html), which was a nationwide study in Japan [12]. The JSEPTIC DIC study retrospectively collected data from patients admitted to the ICU for the treatment of sepsis [13] from January 2011 to December 2013, excluding patients younger than 16 years or who developed sepsis after admission to the ICU (the JSEPTIC DIC study used the definitions of sepsis, severe sepsis, and septic shock from [13] as it was performed before publication of the 2016 definitions [1]). The JSEPTIC DIC study was approved by the Institutional Review Boards of all participating hospitals. The requirement for informed consent was waived because of the retrospective nature of the study. Since this database was already anonymized for individual patient data and institutions, the Institutional Review Board waived the need for review of this post hoc study.

Patients were divided into two groups, the closed ICU group (treated in a closed ICU) and the open ICU group (treated in an open ICU). The JSEPTIC DIC study did not demonstrate a clear definition of open ICU or closed ICUs. Each ICU had reported subjective information about their ICU organization model (open/closed/unclassified) at the initiation of the JSEPTIC DIC study. In Japan, the closed ICU is conventionally defined as a unit that transfers all patient care to an intensive care team that directs patient care with primary responsibility for all care and the open ICU is conventionally defined as an ICU where the intensive care team provides expertise via elective or mandatory consultation without assuming primary responsibility for patient care [14]. Patients treated in an ICU which could not clearly be classified as the closed or open were excluded from the final study population.

### Exposure and outcome variables

The present study used all variables collected in the JSEPTIC DIC study. Variables for which the proportion of missing data was above 10% (fibrinogen, fibrin/fibrinogen degradation products, D-dimer, antithrombin, and lactate) and primary infection site are not included. The main exposure variable was set as the closed ICU or open ICU. The primary outcome measure was survival at discharge. Due to the healthcare system in Japan, no patients were discharged to hospice care.

Covariates for patient characteristics included age, gender, weight, pre-existing organ dysfunction and hemostatic disorders (comorbidity), Acute Physiology and Chronic Health Evaluation (APACHE) II score [15] (day 1), Sequential Organ Failure Assessment (SOFA) score [16] (day 1), systemic inflammatory response syndrome score [17] (days 1), the identification of microorganisms responsible for sepsis, blood culture results, and results of laboratory tests on day 1 including white blood cell count, platelet count, hemoglobin level, and prothrombin time-international normalized ratio (PT-INR). Treatment variables reviewed included administration of medications, including anti-disseminated intravascular coagulation (DIC) or anti-thrombotic drugs, immunoglobulins, low-dose steroids, and transfusion of blood products during the first week after ICU admission. Other therapeutic interventions reviewed included renal replacement therapy, renal replacement therapy for non-renal indications, plasma exchange, polymyxin B direct hemoperfusion (PMX-DHP), extracorporeal membrane oxygenation (ECMO), and use of the intra-aortic balloon pump during the first week after ICU admission.

### Statistical analysis

Baseline characteristics were compared before and after the follow-up period for patients in both the closed and open ICU groups. Distributed continuous variables without a normal distribution are presented as median with interquartile range (IQR). Categorical data are summarized using numbers or percentages. The Mann-Whitney *U* test was used for comparing continuous variables, and the Fisher’s exact test was used for categorical data. As for the main result, multiple logistic regression analysis was performed to estimate the association between the closed/open ICU and survival at discharge from the hospital, adjusted by baseline patient characteristics and treatment variables. As a sensitivity analysis, propensity score matching analysis including the same covariate was performed. For propensity score matching analysis, we use calipers of width equal to 0.1 of the standard deviation. As for sub-analysis, multiple linear regression analysis for the log-transformed values of the length of ICU stay and hospital stay in survived patients was performed with adjustments for the same covariates, and estimated the ratio of the length of stay between the closed ICU and open ICU. The level of significance was set at *p* < 0.05. All statistical analyses were performed with SAS (Version 9.4, SAS Institute Inc., Cary, North Carolina, USA).

## Results

**Fig. 1:**
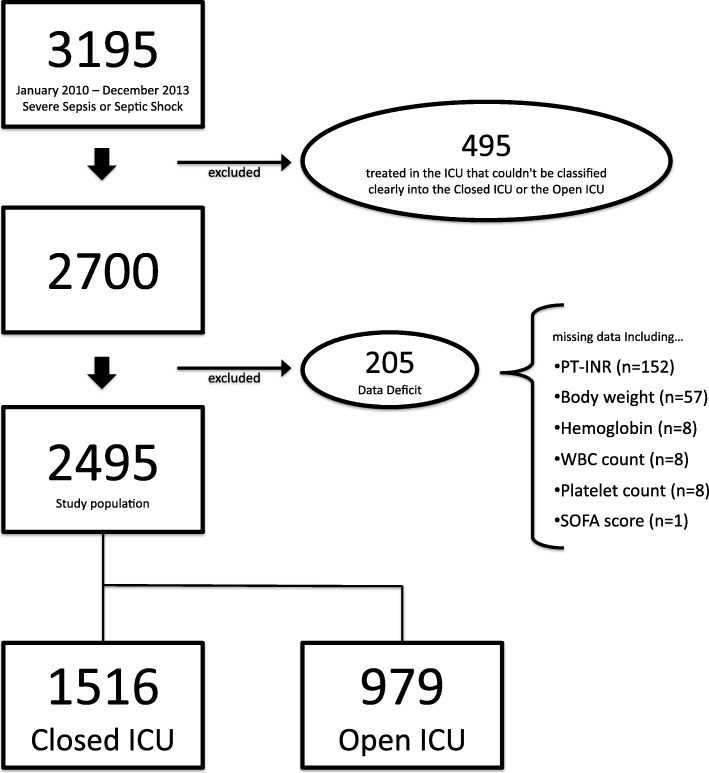
Patient inclusion flow chart. Data for 3195 patients were reviewed and 495 patients did not meet inclusion criteria. There were data deficits for 205 patients, leaving a final study group of 2495 patients. The number of patients with missing data is not considered regarding duplication. PT-INR: prothrombin time international normalized ratio; WBC: white blood cell; SOFA score: Sequential Organ Failure Assessment score

A total of 3195 patients were included in the JSEPTIC DIC study, and 2700 patients were enrolled in this study after excluding patients treated in ICUs not clearly classified as closed or open. There were data deficits in 205 patients, and data for the remaining 2495 patients were analyzed (Fig. 1). The mean age was 72 years, 59.7% male, the median APACHE II score was 23 [17–29], and the median SOFA score (day 1) was 9 [7–12]. The rate of survival at discharge was 66.8% (Table 1). Participating ICUs included 17 (49%) closed and 18 (51%) open.

**Table 1:** Comparison for patient characteristics and treatments (*n* = 2495)

	All patients (*n* = 2495)	Closed ICU (*n* = 1516)	Open ICU (*n* = 979)	*p* value
In-hospital surgical ICU	50 (2.0%)	50 (3.3%)	0 (0.0%)	< 0.001*
In-hospital general ICU	1168 (46.8%)	644 (42.5%)	5234 (53.5%)	< 0.001*
Emergency ICU	1277 (51.2%)	822 (54.2%)	455 (46.5%)	< 0.001*
Number of beds (IQR†)	12 [8–18]	10 [6–18]	12 [10–20]	< 0.001*
SOFA score (IQR†)	9 [7–12]	9 [7–13]	9 [6–12]	0.004^*^
APACHE II score (IQR†)	22 [17–29]	23 [18–29]	21 [16–28]	< 0.001*
SIRS score (IQR†)	3 [2–4]	3 [2–4]	3 [2–4]	0.058
Age (IQR†, year old)	72 [62–80]	72 [62–80]	73 [63–81]	0.007*
Sex (male, %)	1490 (59.7%)	916 (60.4%)	574 (58.6%)	0.373
Body weight (IQR†, kg)	54.5 [46.3–64]	54.7 [46.5–65]	54.0 [46.0–63]	0.208
White blood cell count (IQR†, × 10^3^)	11.2 [4.5–17.8]	11.00 [4.40–17.76]	11.66 [4.85–17.80]	0.199
Hemoglobin (IQR†, g/dl)	10.6 [9–12.4]	10.5 [8.9–12.4]	10.6 [9.1–12.5]	0.055
Platelet count (IQR†, × 10^3^)	120 [64–191]	121 [64–194]	118 [64–186]	0.498
Prothrombin time international normalize ratio (IQR†)	1.34 [1.17–1.61]	1.35 [1.18–1.63]	1.32 [1.16–1.56]	0.057
Co-morbidities				
Liver failure (yes, %)	104 (4.2%)	68 (4.5%)	36 (3.7%)	0.324
Respiratory failure (yes, %)	98 (3.9%)	64 (4.2%)	34 (3.5%)	0.347
Cardiac failure (yes, %)	134 (5.4%)	82 (5.4%)	52 (5.3%)	0.916
Renal failure (yes, %)	217 (8.7%)	114 (7.5%)	103 (10.5%)	0.009*
Immunological disorder (yes, %)	381 (15.3%)	263 (17.3%)	118 (12.1%)	< 0.001*
Hematologic disorder				
Cirrhosis (yes, %)	97 (3.9%)	63 (4.2%)	34 (3.5%)	0.389
Hematologic malignancy (yes, %)	84 (3.4%)	58 (3.8%)	26 (2.7%)	0.113
Chemotherapy (yes, %)	114 (4.6%)	79 (5.2%)	35 (3.6%)	0.056
Warfarin intake (yes, %)	113 (4.5%)	73 (4.8%)	40 (4.1%)	0.392
Others (yes, %)	48 (1.9%)	33 (2.2%)	15 (1.5%)	0.252
Positive blood culture	1111 (44.5%)	679 (44.8%)	432 (44.1%)	0.745
Negative blood culture	1240 (49.7%)	778 (51.3%)	462 (47.2%)	0.044*
No blood culture	144 (5.8%)	59 (3.9%)	85 (8.7%)	< 0.001*
Viral infection	22 (0.9%)	18 (1.2%)	4 (0.4%)	0.042*
GNR infection	912 (36.6%)	528 (34.8%)	384 (39.2%)	0.026*
GPC infection	586 (23.5%)	344 (22.7%)	242 (24.7%)	0.243
Fungal infection	38 (1.5%)	27 (1.8%)	11 (1.1%)	0.190
Mixed infection	339 (13.6%)	246 (16.2%)	93 (9.5%)	< 0.001*
Other infection	45 (1.8%)	33 (2.2%)	12 (1.2%)	0.081
Unknown infection	553 (22.2%)	320 (21.1%)	233 (23.8%)	0.114
Red blood cell transfusion (IQR†, units)	0 [0–4]	0 [0–4]	0 [0–4]	< 0.001*
Fresh frozen plasma transfusion (IQR†, units)	0 [0–4]	0 [0–5]	0 [0–4]	0.002*
Platelet concentration transfusion (IQR†, units)	0 [0–0]	0 [0–10]	0 [0–0]	< 0.001*
Treatment for DIC (yes, %)	1074 (43.0%)	656 (43.3%)	418 (42.7%)	0.777
Antithrombin III (yes, %)	726 (29.1%)	447 (29.5%)	279 (28.5%)	0.596
rhsTM (yes, %)	636 (25.5%)	420 (27.7%)	216 (22.1%)	0.002*
Nafamostat (yes, %)	880 (35.3%)	533 (35.2%)	347 (35.5%)	0.884
Heparin (yes, %)	453 (18.2%)	247 (16.3%)	206 (21.0%)	0.003*
Warfarin (yes, %)	39 (1.6%)	15 (1.0%)	24 (2.5%)	0.004*
Antiplatelet (yes, %)	56 (2.2%)	37 (2.4%)	19 (1.9%)	0.411
Others (yes, %)	15 (0.6%)	7 (0.5%)	8 (0.8%)	0.262
Specific treatment				
Immunoglobulin (yes, %)	743 (29.8%)	447 (29.5%)	296 (30.2%)	0.689
Low dose steroid (yes, %)	600 (24.0%)	413 (27.2%)	187 (19.1%)	< 0.001*
Renal replacement therapy (yes, %)	664 (26.6%)	452 (29.8%)	212 (21.7%)	< 0.001*
Renal replacement therapy not for renal indication (yes, %)	165 (6.6%)	58 (3.8%)	107 (10.9%)	< 0.001*
Polymyxin B direct hemoperfusion (yes, %)	547 (21.9%)	316 (20.8%)	231 (23.6%)	0.105
Plasma exchange (yes, %)	20 (0.8%)	9 (0.6%)	11 (1.1%)	0.147
Veno-arterial ECMO (yes, %)	23 (0.9%)	20 (1.3%)	3 (0.3%)	0.010*
Veno-venus ECMO (yes, %)	27 (1.1%)	26 (1.7%)	1 (0.1%)	< 0.001*
Intra-aortic balloon pumping (yes, %)	11 (0.4%)	7 (0.5%)	4 (0.4%)	0.845
Mechanical ventilation support (yes, %)	1799 (72.1%)	1184 (78.1%)	615 (62.8%)	< 0.001*
Survival discharge (yes, %)	1668 (66.9%)	1014 (66.9%)	654 (66.8%)	0.965

*IQR†* median [25%, 75%] for continuous variables, *ICU* intensive care unit, *SOFA score* Sequential Organ Failure Assessment score, *APACHE II score* Acute Physiology and Chronic Health Evaluation II score, *SIRS score* systemic inflammatory response syndrome score, *GNR* gram-negative rods, *GPC* gram-positive coccus, *DIC* disseminated intravascular coagulation, *rhsTM* recombinant human soluble thrombomodulin, *ECMO* extracorporeal membrane oxygenation

*p < 0.05

There were 979 patients treated in open ICUs and 1516 patients in closed ICUs. A comparison of the characteristics of these two groups is shown in Table 1. The closed ICU group included fewer patients with renal failure than the open ICU group (closed vs open = 7.5% vs 10.5%, *p* = 0.009), and the closed ICU group included significantly more patients with immunological disorders (closed vs open = 17.4% vs 12.1%, *p* < .001). The APACHE II and SOFA scores (day 1) of patients in the closed ICU group is significantly higher than that in the open ICU (APACHE II score: closed vs open = 23 [18–29] vs 21 [16–28], *p* < 0.001, SOFA score: closed vs open = 9 [7–13] vs 9 [6–12], *p* = 0.004). Patients in the closed ICU group were more severely ill than patients in the open ICU group, based on these scores.

The value of variables examined was different comparing treatments used during follow-up in the closed and open groups. Recombinant human soluble thrombomodulin (rhsTM) is used more frequently in the closed ICU group (closed vs open = 27.7% vs 22.1%, *p* = 0.002), but heparin and warfarin are used more often in the open ICU group (heparin; closed vs open = 16% vs 21%, *p* = 0.003), (warfarin; closed vs open = 1.0% vs 2.5%, *p* = 0.004). The use of low-dose steroids in patients with sepsis (closed vs open = 27.2% vs 19.1%, *p* < 0.001) and renal replacement therapy was more frequent in the closed ICU group (closed vs open = 29.8% vs 21.7%, *p* < 0.001), but renal replacement therapy for non-renal indications was more often performed in the open ICU group (closed vs open = 3.8% vs 10.9%, *p* < 0.001). Mechanical ventilation was more frequently used in the closed ICU group (closed vs open = 78.1% vs 62.8%, *p* < 0.001).

In the main analysis, the crude, the unadjusted analysis did not show a significant difference in survival at discharge between the closed and open ICU models (closed vs open = 66.9% vs 66.8%, *p* = 0.97). However, in multiple logistic regression analysis adjusted by baseline, patient characteristics and treatment variables had a significant association between treatment in a closed ICU and survival at discharge (odds ratio = 1.59, 95% CI [1.276–1.827], *p* = 0.001) (Table 2). In sensitivity analysis (702 pairs after propensity score matching), the closed ICU group showed a significantly higher survival rate, compared to the open ICU (71.8% vs 65.2%, odds ratio = 1.41 (95% CI [1.12–1.77]), *p* = 0.011) (Table 3, Additional file 1: Table S1).

**Table 2:** Multiple logistic regression analysis adjusted for baseline patient characteristics and treatment (*n* = 2495)

		Coefficient	Adj OR	95% CI			*p* value
Closed ICU	(Ref: open ICU)	0.463	1.589	1.276	–	1.827	0.001*
Type of ICU	(Ref: in-hospital general ICU)						
In-hospital surgical ICU		− 0.525	0.592	0.286	–	1.223	0.157
Emergency ICU		0.101	1.106	0.9	–	1.36	0.338
Number of beds	(Continuous)	− 0.002	0.998	0.981	–	1.016	0.846
Blood culture	(Ref: no blood culture)						
Positive		− 0.785	0.456	0.279	–	0.746	0.002*
Negative		− 0.407	0.666	0.419	–	1.059	0.086
Infection type	(Ref: unknown infection)						
Viral infection		0.447	1.563	0.48	–	5.085	0.458
GNR infection		0.606	1.833	1.349	–	2.492	< 0.001*
GPC infection		0.224	1.251	0.898	–	1.743	0.185
Fungal infection		− 0.490	0.613	0.284	–	1.321	0.211
Mixed infection		0.015	1.015	0.715	–	1.442	0.933
Other infection		0.656	1.926	0.908	–	4.086	0.088
SOFA score	(Continuous)	− 0.133	0.876	0.845	–	0.908	< 0.001*
APACHE II score	(Continuous)	− 0.018	0.982	0.969	–	0.996	0.010*
SIRS score	(Continuous)	− 0.115	0.892	0.796	–	0.999	0.048*
Sex, female	(Ref: male)	0.373	1.451	1.171	–	1.799	< 0.001*
Age	(Continuous)	− 0.0238	0.976	0.969	–	0.985	< 0.001*
Body weight	(Continuous)	0.0193	1.020	1.011	–	1.028	< 0.001*
White blood cell count (× 103)	(Ref: 3.5~9)						
0~3.5		0.267	1.306	0.741	–	2.302	0.355
9~		− 1.407	0.245	0.019	–	3.107	0.278
Hemoglobin	(Continuous)	0.049	1.05	1.006	–	1.097	0.026*
Platelet count (per 104)	(Continuous)	0.003	1.003	0.991	–	1.015	0.624
PT-INR	(Continuous)	− 0.0614	0.94	0.868	–	1.019	0.135
Co-morbidities	(Ref: no)						
Liver failure		− 0.724	0.485	0.288	–	0.817	0.007*
Respiratory failure		− 0.434	0.648	0.404	–	1.038	0.071
Cardiac failure		− 0.486	0.615	0.403	–	0.938	0.024*
Renal failure		− 0.640	0.527	0.368	–	0.756	< 0.001*
Immunological disorder		− 0.547	0.579	0.43	–	0.779	< 0.001*
Hematologic disorder	(Ref: no)	− 0.355	0.701	0.516	–	0.952	0.023*
Red blood cell transfusion	(Continuous)	− 0.022	0.978	0.956	–	1.001	0.061
Fresh frozen plasma transfusion	(Continuous)	− 0.010	0.99	0.98	–	1.001	0.083
Platelet concentration transfusion	(Continuous)	0.002	1.002	0.996	–	1.008	0.529
Treatment for DIC	(Ref: no)						
Antithrombin III		0.185	1.203	0.938	–	1.544	0.145
rhTM		0.294	1.341	1.043	–	1.725	0.022*
Nafamostat		0.152	1.165	0.869	–	1.561	0.308
Heparin		0.334	1.396	1.06	–	1.839	0.018*
Warfarin		− 0.148	0.862	0.400	–	1.861	0.706
Antiplatelet		0.318	1.374	0.679	–	2.778	0.377
Others		0.303	1.354	0.318	–	5.768	0.682
Specific treatment	(Ref: no)						
Immunoglobulin		0.147	1.158	0.909	–	1.476	0.236
Low-dose steroid		− 0.534	0.586	0.462	–	0.744	< 0.001*
Renal replacement therapy		− 0.390	0.677	0.495	–	0.926	0.015*
Renal replacement therapy not for renal indication		− 0.279	0.756	0.496	–	1.153	0.195
Polymyxin B direct hemoperfusion		0.365	1.44	1.095	–	1.894	0.009*
Plasma exchange		− 0.457	0.633	0.189	–	2.118	0.459
Veno-arterial ECMO		− 2.475	0.084	0.021	–	0.341	< 0.001*
Veno-venus ECMO		− 1.169	0.311	0.111	–	0.865	0.025*
Ventilation support		− 0.815	0.443	0.339		0.578	< 0.001*
Intra-aortic balloon pumping		0.993	2.699	0.467	–	15.608	0.268

White blood cell count (× 103)

*adj OR* adjusted odds ratio, *CI* confidence intervals, *ICU* intensive care unit, *GNR* gram-negative rods, *GPC* gram-positive coccus, *SOFA score* Sequential Organ Failure Assessment score, *APACHE II score* Acute Physiology and Chronic Health Evaluation II score, *SIRS score* systemic inflammatory response syndrome score, *PT-INR* prothrombin time-international normalized ratio, *DIC* disseminated intravascular coagulation, *rhsTM* recombinant human soluble thrombomodulin, *ECMO* extracorporeal

*p < 0.05

**Table 3:** Cross table after propensity score matching

	Open ICU	Closed ICU	
Death	244	198	442
Survive	458	504	962
	702	702	1404

*P* = 0.011 (chi-squared test). OR = 1.41 (95% CI 1.12–1.77)

*OR* odds ratio, *CI* confidence intervals

In sub-analysis, multiple linear regression analysis using the data about the length of ICU stay and hospital stay as an objective variable showed that the length of ICU stay of patients treated in closed ICUs was significantly shorter (20% less) than patients in open ICUs, but this significance was not seen in the length of hospital stay (Table 4).

**Table 4:** The multiple liner regression analysis for the log-transformed values of the length of ICU stay and hospital stay in survived patients

	Ratio	95% confidence interval		*p* value
Sub-analysis 1: multiple linear regression in survived patients for the length of ICU stay				
(Closed ICU/open ICU) *	0.80	0.75	0.87	< 0.001
Sub-analysis 2: multiple linear regression in survived patients for the length of hospital stay				
(Closed ICU/open ICU) *	0.98	0.90	1.07	0.642

*Adjusted by both patient backgrounds and treatment variables (exactly the same covariates in main analysis)

## Discussion

In this analysis of a large nationwide Japanese cohort of patients with sepsis and septic shock, patient management in a closed ICU is significantly associated with improved rate of survival at discharge and a decrease in the length of ICU stay.

### Efficacy of the treatment of sepsis in a closed ICU

According to multiple logistic regression analysis adjusted for baseline characteristics and treatment variables, treatment in a closed ICU had a significant association with improved survival at discharge (odds ratio = 1.59, 95% CI [1.276–1.827], *p* = 0.001) (Table 2). This result suggests that the efficacy of care in a closed ICU may depend on the management of sepsis conducted in a closed ICU. However, according to the crude comparison (Table 1), the patient cohorts were different in the closed and open ICUs. We additionally performed propensity score matching analysis as a sensitivity analysis, and the closed ICU group has a significantly higher survival rate, compared to open ICU (Table 3, Additional file 1: Table S1). The result of the main analysis is fully supported by the sensitivity analysis. There is a significant association between treatment in a closed ICU and improved survival at discharge.

A significant difference in the rate of using various treatment modalities in closed and open ICUs is identified in this study (Table 1). Although these treatments are adjuvant therapies for sepsis and their efficacy is controversial, the difference in the rate of using these treatments performed in each ICU may be partly responsible for the observed significant association between treatment in a closed ICU and survival at discharge. The present study demonstrates a significant association between prognosis and each specific treatment for sepsis including use of recombinant human soluble thrombomodulin (rhsTM), low-dose steroid therapy, or PMX-DHP.

Some DIC treatment guidelines recommend the use of rhsTM rather than heparin for patients with DIC due to sepsis [18], and both positive and negative evidence regarding the survival benefit of rhsTM have been reported [19, 20]. The result of a phase 3 trial of rhsTM [21] is awaited. The JSEPTIC DIC study using propensity score analysis reported a survival benefit with the use of rhsTM [12]. The present study, which uses the same data as the JSEPTIC DIC study, also shows a significant association between survival at discharge and rhsTM, using a different statistical analysis, and the use of rhsTM was more frequent in closed ICUs and the use of heparin was more frequent in open ICUs. This difference may be partly responsible for the association between improved survival at discharge and treatment in a closed ICU.

The efficacy of steroid therapy to reduce mortality in patients with sepsis has been controversial [22, 23]. In the present study, the use of low-dose steroids (which is more frequent in a closed ICU) was significantly associated with in-hospital mortality, but the route of administration, timing, and presence of side effects were not reviewed. A significant association between the use of PMX-DHP and survival at discharge was also shown, but there was no significant difference of the using rate of PMX-DHP between the closed and open ICUs. Some trials showed a survival benefit with the use of PMX-DHP [24], but others have not [25]. The effect of PMX-DHP remains controversial, and the results of another prospective multicenter randomized controlled trial is awaited, including a phase 3 trial of PMX-DHP [26].

We consider that a significant association between treatment in a closed ICU and survival at discharge can be related to the high-quality care provided in a closed ICU. A high rate of compliance with guidelines is one element of “high quality” intensive care. This element affects the patients’ prognosis, and compliance is different in open and closed ICUs. Some reports show that patients with acute respiratory distress syndrome (ARDS) cared for in a closed ICU had lower hospital mortality due to the increased rate of compliance with guideline-recommended lung-protective ventilation [27] (a safety management was given for the patients with ARDS in the closed ICU). Considering the present study, a significantly lower rate of obtaining blood cultures (blood cultures are strongly recommended in the sepsis guideline [5, 6]) in the open ICU (Table 1) suggests that compliance with the guidelines in an open ICU may be lower than that in closed ICUs and the lower compliance may contribute to the higher mortality in open ICUs. Patient with sepsis commonly need ICU care, and in this decade, guidelines for the treatment of sepsis have been published from international societies [5]. The management of patients with sepsis has been qualified and standardized throughout the world, and the closed ICU can provide standardized intensive care for patients with sepsis based on the guidelines, which can improve the outcomes [28].

The safety management, prevention, or rapid and optimal response to complications is an element of “high quality” intensive care. Another report from the Leapfrog Group reported that applying ICU physician safety staffing standards could save more than 54,000 lives in the USA each year [29]. Intensivists can be an expert in safety management and complication management (prevention or appropriate response) in the ICU. Although the present study did *not* investigate the differences in incidence of complications or other safety management issues between closed and open ICUs, this potential difference may contribute to the significant association between treatment in a closed ICU and a higher survival rate at discharge. Future study should be focused on the differences in these factors.

### Study limitations

This is a sub-analysis of the JSEPTIC DIC study, which used retrospective data. The 205 patients excluded because of data insufficiency may have an impact on the results although these excluded patients represent less than 10% of the study population. The J-SEPTIC DIC study did not clearly define open and closed ICUs, and each ICU had reported subjective information about their ICU model (open/closed/unclassified). This study comparing the outcomes of patients with sepsis in open and closed ICUs was conducted based on a definition that was subjectively reported from each institution. Although the present study demonstrates the potential of improved efficacy of the treatment of sepsis in a closed ICU, this result may be unreliable because of an unclear definition of the types of ICUs. Further study should clearly define open and closed ICUs to investigate the association between the outcome of patients with sepsis and ICU organization models.

This study focuses only on the association between outcomes and the type of ICU model. Details of differences in ICU treatment and quality of care in each institution have not been analyzed. Although many kinds of ICU staffing components influence ICU outcomes, the information such as hospital characteristics, general/university; the staffing numbers for open or closed ICU’s in this study, nursing/junior doctors/fellows/certificated specialist intensivists/non-certificated intensivist; or nighttime coverage by certificated specialist intensivists was not available in this *post hoc* analysis. These staffing patterns must influence the outcomes of patients in the ICU, but this information was unavailable in this study. This is a severe limitation of the present study. Future studies should include staffing information to investigate a relationship between the outcomes of patients with sepsis and treatment using each ICU model (open/closed).

Although many kinds of guidelines or safety management protocols in the ICU such as hand hygiene protocols, ventilation-associated pneumonia bundles, and catheter-related blood stream infection management impact outcomes in the care of patients with sepsis, compliance with these care bundles was not evaluated in this study. Future studies should focus on compliance with sepsis and other care guidelines, treatment differences, and quality of care in the ICU. To further define the benefit of a closed ICU, additional prospective multicenter studies are warranted.

## Conclusions

This nationwide retrospective *post hoc* analysis of patients with sepsis in Japan shows a significant association between treatment in a closed ICU and improved survival at discharge, although there are acknowledged study limitations. Future prospective trials are indispensable to evaluate the efficacy of treatment in a closed ICU for the treatment of patients with sepsis. These studies should define the ICU models (closed/open) clearly and focus on staffing patterns, compliance with care guidelines, treatment differences, and quality of care in ICUs with both closed and open ICU models.

## Additional file

Additional file 1: **Table S1.** Comparison for patient characteristics and treatments after matching. (DOCX 25 kb)

## Abbreviations

APACHE II score: Acute Physiology and Chronic Health Evaluation II score
ARDS: Acute respiratory distress syndrome
DIC: Disseminated intravascular coagulation
ECMO: Extracorporeal membrane oxygenation
ICU: Intensive care unit
JSEPTIC DIC study: Japan Septic Disseminated Intravascular Coagulation study
PMX-DHP: Polymyxin B direct hemoperfusion
PT-INR: Prothrombin time-international normalized ratio
SOFA score: Sequential Organ Failure Assessment score
