# Supplementary material for: Treatment of patients with sepsis in a closed intensive care unit is associated with improved survival: a nationwide observational study in Japan
Source: J Intensive Care. 2018 Sep 3;6:57. doi: 10.1186/s40560-018-0322-8 (PMC6122219; doi:10.1186/s40560-018-0322-8)
Supplement: Supplementary file 1 — Table S1. Comparison for patient characteristics and treatments after matching. (DOCX 25 kb) [file 40560_2018_322_MOESM1_ESM.docx]

Additional file 1: Table S1 Comparison for patient characteristics and treatments after matching

|  | | Closed ICU (n=702) | Open ICU (n=702) | P-Value |
| --- | --- | --- | --- | --- |
| In-hospital Surgical ICU | | 1 (0.1 %) | 0 (0.0 %) | 1.000 |
| In-hospital General ICU | | 342 (48.7 %) | 356 (50.7 %) | 0.478 |
| Emergency ICU | | 359 (51.1 %) | 346 (49.3 %) | 0.513 |
| Number of Beds (IQR†) | | 12 [10, 18] | 10 [8, 18] | 0.002* |
| SOFA score (IQR†) | | 9 [6, 12] | 9 [6, 12] | 0.940 |
| APACHE II score (IQR†) | | 21 [16, 28] | 22 [16, 29] | 0.677 |
| SIRS score (IQR†) | | 3 [2, 4] | 3 [2, 4] | 0.926 |
| age (IQR†, year-old) | | 73 [62, 81] | 73 [62, 81] | 0.508 |
| sex (male, %) | | 405 (57.7 %) | 420 (59.8 %) | 0.460 |
| body weight (IQR†, kg) | | 53.0 [45.2, 63.2] | 54.6 [46.0, 63.0] | 0.456 |
| white blood cell count (IQR†, ×10^3^) | | 11.6 [5.4, 18.6] | 11.5 [4.9, 18.0] | 0.436 |
| hemoglobin (IQR†, g/dl) | | 10.7 [8.9, 12.2] | 10.4 [8.9, 12.3] | 0.910 |
| platelet count (IQR†, × 10^3^) | | 120 [64, 192] | 120 [64, 190] | 0.842 |
| prothrombin time international normalize ratio (IQR†) | | 1.35 [1.17, 1.65] | 1.31 [1.16, 1.55] | 0.054 |
| co-morbidities | |  |  |  |
|  | liver failure (yes, %) | 28 (4.0 %) | 33 (4.7 %) | 0.583 |
|  | respiratory failure (yes, %) | 23 (3.3 %) | 30 (4.3 %) | 0.410 |
|  | cardiac failure (yes, %) | 43 (6.1 %) | 30 (4.3 %) | 0.149 |
|  | renal failure (yes, %) | 64 (9.1 %) | 65 (9.3 %) | 1.000 |
|  | immunological disorder (yes, %) | 106 (15.1 %) | 108 (15.4 %) | 0.940 |
|  | hematologic disorder |  |  |  |
|  | cirrhosis (yes, %) | 30 (4.3 %) | 31 (4.4 %) | 1.000 |
|  | hematologic malignancy (yes, %) | 19 (2.7 %) | 17 (2.4 %) | 0.868 |
|  | chemotherapy (yes, %) | 29 (4.1 %) | 31 (4.4 %) | 0.896 |
|  | warfarin intake (yes, %) | 43 (6.1 %) | 24 (3.4 %) | 0.023* |
|  | others (yes, %) | 11 (1.6 %) | 13 (1.9 %) | 0.831 |
|  | Positive blood culture | 295 (42.0 %) | 312 (44.4 %) | 0.392 |
|  | Negative blood culture | 358 (51.0 %) | 353 (50.3 %) | 0.830 |
|  | No blood culture | 49 (7.0 %) | 37 (5.3 %) | 0.219 |
|  | Viral infection | 5 (0.7 %) | 3 (0.4 %) | 0.724 |
|  | Gram Negative Rods infection | 260 (37.0 %) | 254 (36.2 %) | 0.781 |
|  | Gram Positive Coccus infection | 168 (23.9 %) | 178 (25.4 %) | 0.574 |
|  | Fungal infection | 10 (1.4 %) | 10 (1.4 %) | 1.000 |
|  | Mixed infection | 88 (12.5 %) | 87 (12.4 %) | 1.000 |
|  | Others infection | 9 (1.3 %) | 10 (1.4 %) | 1.000 |
|  | Unknown infection | 162 (23.1 %) | 160 (22.8 %) | 0.949 |
| red blood cell transfusion (IQR†, units) | | 0 [0, 4] | 0 [0, 4] | 0.246 |
| fresh frozen plasma transfusion (IQR†, units) | | 0 [0, 4] | 0 [0, 4] | 0.374 |
| platelet concentration transfusion (IQR†, units) | | 0 [0, 0] | 0 [0, 0] | 0.817 |
| treatment for DIC (yes, %) | | 285 (40.6 %) | 296 (42.2 %) | 0.586 |
|  | antithrombin III (yes, %) | 182 (25.9 %) | 196 (27.9 %) | 0.434 |
|  | rhsTM (yes, %) | 172 (24.5 %) | 173 (24.6 %) | 1.000 |
|  | nafamostat (yes, %) | 231 (32.9 %) | 229 (32.6 %) | 0.955 |
|  | heparin (yes, %) | 129 (18.4 %) | 127 (18.1 %) | 0.946 |
|  | warfarin (yes, %) | 12 (1.7 %) | 9 (1.3 %) | 0.663 |
|  | antiplatelet (yes, %) | 14 (2.0 %) | 18 (2.6 %) | 0.584 |
|  | others (yes, %) | 4 (0.6 %) | 5 (0.7 %) | 1.000 |
| specific treatment | |  |  |  |
|  | immunoglobulin (yes, %) | 193 (27.5 %) | 202 (28.8 %) | 0.640 |
|  | low dose steroid (yes, %) | 161 (22.9 %) | 157 (22.4 %) | 0.848 |
|  | renal replacement therapy (yes, %) | 161 (22.9 %) | 173 (24.6 %) | 0.468 |
|  | renal replacement therapy not for renal indication (yes, %) | 39 (5.6 %) | 26 (3.7 %) | 0.118 |
|  | polymyxin B direct hemoperfusion (yes, %) | 139 (19.8 %) | 144 (20.5 %) | 0.789 |
|  | plasma exchange (yes, %) | 7 (1.0 %) | 4 (0.6 %) | 0.546 |
|  | veno-arterial ECMO (yes, %) | 2 (0.3 %) | 3 (0.4 %) | 1.000 |
|  | veno-venus ECMO (yes, %) | 3 (0.4 %) | 1 (0.1 %) | 0.480 |
|  | intra-aortic balloon pumping (yes, %) | 3 (0.4 %) | 3 (0.4 %) | 1.000 |
| survival discharge (yes, %) | | 504 (71.8 %) | 458 (65.2 %) | 0.011* |
| IQR†; median [25%, 75%] for continuous variables | | | | |
| *; p<0.05 | | | | |
| ICU; intensive Care Units | | | | |
| DIC; disseminated intravascular coagulation | | | | |
| rhsTM; recombinant human soluble thrombomodulin | | | | |
| SOFA score; Sequential Organ Failure Assessment score | | | | |
| APACHE Ⅱ score; Acute Physiology and Chronic Health Evaluation II score | | | | |
| SIRS score; systemic inflammatory response syndrome score | | | | |
| ECMO; extracorporeal membrane oxygenation | | | | |
